# Bilateral Symmetrical Fusion of Permanent Mandibular Lateral Incisors and Canines: A Rare Clinical Case

**DOI:** 10.1002/ccr3.71835

**Published:** 2026-01-11

**Authors:** Suresh Kandagal Veerabhadrappa, Jayanth Kumar Vadivel, Mohamed Zahoor Ul Huqh, Siddharthan Selvaraj, Anand Marya, Seema Yadav

**Affiliations:** ^1^ Faculty of Dentistry SEGi University, Petaling Jaya Petaling Jaya Selangor Malaysia; ^2^ Saveetha Dental College and Hospitals, Saveetha University Chennai India; ^3^ Faculty of Dentistry University of Puthisastra Phnom Penh Cambodia; ^4^ Dr. D. Y. Patil Dental College & Hospital, Dr. D. Y. Patil Vidyapeeth (Deemed to Be University) Pune India

**Keywords:** anomaly, canine, dentition, developmental, fusion, gene, incisor, permanent, tooth

## Abstract

Fusion is a rare developmental anomaly characterized by the union of two adjacent tooth germs during the morphodifferentiation stage of tooth formation, resulting in a single enlarged tooth. Its prevalence ranges from 0.4% to 0.9% in the primary dentition and approximately 0.2% in the permanent dentition. Unilateral fusion is more commonly observed in the primary dentition compared to the permanent dentition. Bilateral fusion in permanent teeth is extremely rare, with a reported prevalence of only 0.05%, most frequently affecting the incisors. Bilateral fusion involving the mandibular permanent lateral incisors and canines is even more uncommon. Due to its low prevalence in the permanent dentition, the clinical significance, diagnosis, and radiographic presentation of such anomalies are poorly understood. This paper highlights an exceptionally rare case of bilateral symmetrical fusion of the permanent mandibular lateral incisors and canines, which led to dental crowding and challenges in maintaining effective plaque control. To the best of our knowledge, this specific type of bilateral fusion has not been previously reported in the literature.

## Key Clinical Message

This rare clinical case gives the importance of morphological and radiological investigation ensuring optimal aesthetic and functional outcome.

## 1 Introduction

Fusion of teeth is characterized by the joining of two or more separate tooth buds during odontogenesis, affecting both the number and size of teeth [1]. It can be either complete or partial, depending on the developmental stage and degree of calcification. Complete fusion occurs when adjacent tooth buds come into contact before calcification begins [2]. Clinically, this is manifested as an abnormally wide crown, often with a groove separating the mesial and distal portions. Partial fusion occurs after crown calcification and typically involves union at the level of the enamel, enamel and dentin, or the root, resulting in a large bifid crown with either a single pulp chamber or separate pulp chambers and root canals [1, 2, 3].

Bilateral symmetrical fusion of permanent mandibular lateral incisors and canines' prevalence ranges from 0.4% to 0.9% in the primary dentition and approximately 0.2% in the permanent dentition, with unilateral cases being more common than bilateral ones [4]. Anterior teeth are more commonly involved than posterior teeth in both dentitions [5]. The reported prevalence of fused primary teeth varies depending on the population studied, diagnostic criteria, examination methods, and ethnic background [5]. A higher incidence has been observed among Asians and Native Americans [1]; however, no gender differences have been reported. Bilateral fusion in the permanent dentition is extremely uncommon, with a reported prevalence of only about 0.05%, and most often involves maxillary incisors. The limited number of documented cases makes its clinical presentation, diagnosis, and management less well understood. While unilateral and bilateral fusion in the deciduous dentition [6, 7, 8, 9, 10, 11], as well as unilateral fusion involving the permanent dentition [3, 4, 5, 12, 13, 14], have been well documented in the literature, reports of bilateral symmetrical fusion involving permanent teeth are extremely rare.

Previous literature describes fusion in unilateral cases [3, 5] or bilateral cases involving the mandibular central and lateral incisors [4, 14, 15, 16, 17]. To the best of our knowledge, no prior report describes bilateral symmetrical complete fusion of the permanent mandibular lateral incisors and canines. The purpose of this paper is to present this rare entity, highlighting the clinical and radiographic diagnostic approaches, exploring potential etiological factors, and reviewing the literature on the fusion of the permanent mandibular lateral incisor and canine.

## 2 Case History

A 35‐year‐old Malaysian Chinese female patient presented to SEGI University Dental Faculty with a complaint of bleeding gums in the lower front teeth region. Her medical and family history was unremarkable, and there was no reported history of dental trauma. Examination of her parents and siblings revealed no unusual findings and their dentition revealed no abnormalities. Furthermore, the patient's mother did not take any medication and was not exposed to radiation or trauma during pregnancy.

## 3 Differential Diagnosis, Investigations, and Treatment

**FIGURE 1:**
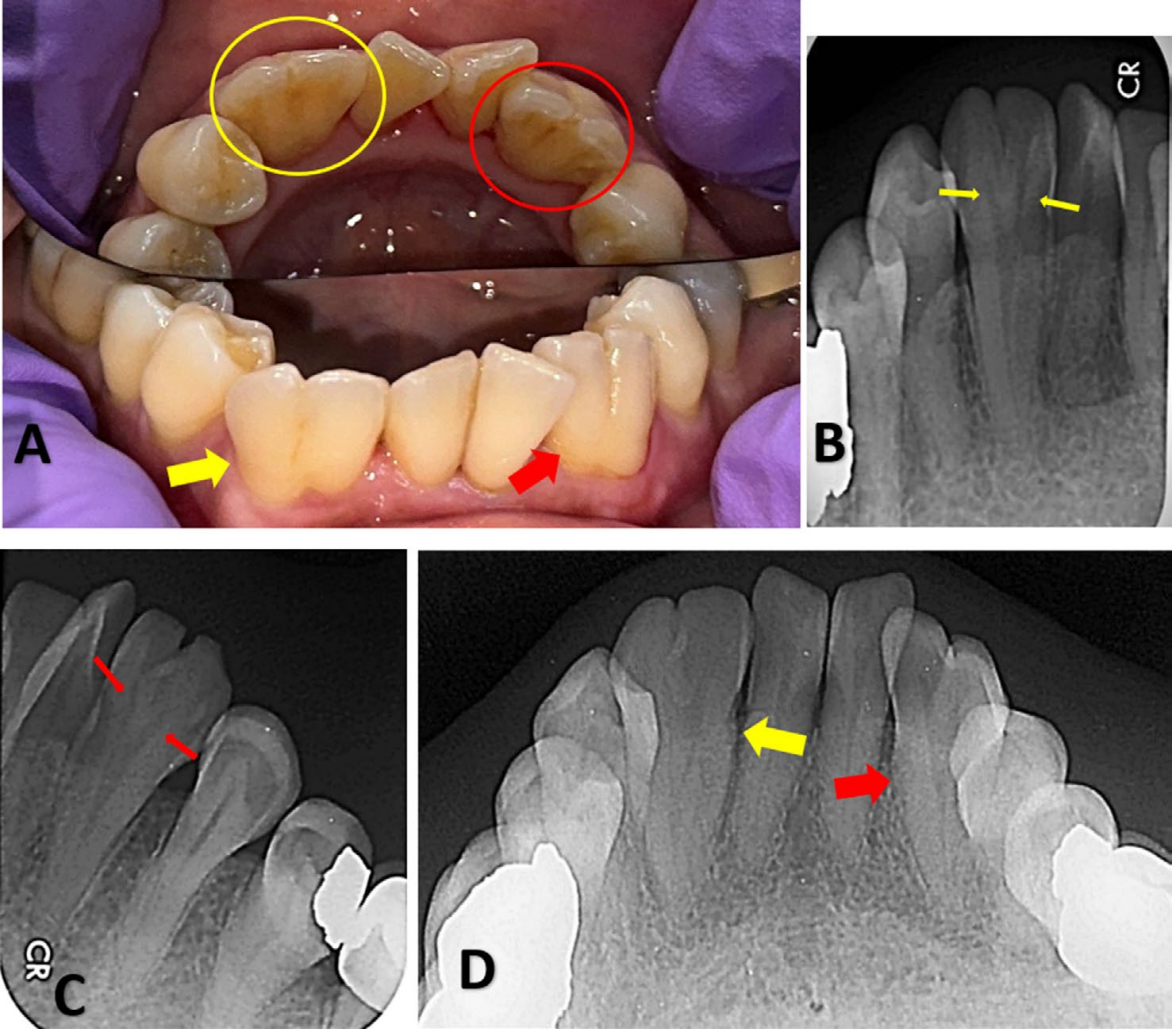
(A) Intraoral photograph showing bilaterally fused mandibular lateral incisors and canines with visible developmental grooves on the labial surfaces and mild tooth surface loss in relation to teeth 42 and 43. (B, C) Intraoral periapical radiographs demonstrating bilateral fusion of the mandibular lateral incisors and canines, with two distinct coronal pulp horns converging into a single pulp chamber at the cervical level. (D) Mandibular occlusal radiograph confirming bilateral fusion of the lateral incisors and canines.

Intraoral examination revealed bilateral fusion of the right and left mandibular lateral incisors and canines, forming a single large crown with a developmental groove on both the labial and lingual aspects, extending into the cervical area (Figure 1A). The fused teeth were free of caries; however, crowding of the central incisors was observed, along with gingival inflammation due to local factors. Mild tooth surface loss was also noted on the right lower fused tooth. No mobility was observed in either of the fused teeth. No other developmental abnormalities were noted in the dentition. All other teeth and the periodontium appeared normal, although the patient exhibited two missing teeth and multiple restorations.

## 4 Conclusion and Results (Outcome and Follow‐Up)

Intraoral periapical radiographs (Figure 1B,C) revealed fused crowns of the mandibular lateral incisors and canines on the right and left side, resulting in an enlarged crown with two small pulpal horns that converged into a single pulp chamber at the cervical level of the crown. Both fused teeth exhibited a single root with a single canal. Mandibular occlusal radiographs (Figure 1D) demonstrated bilaterally fused permanent lateral incisors and canines, forming a single enlarged crown and root structure. Both fused teeth exhibited a similar radiographic presentation.

**FIGURE 2:**
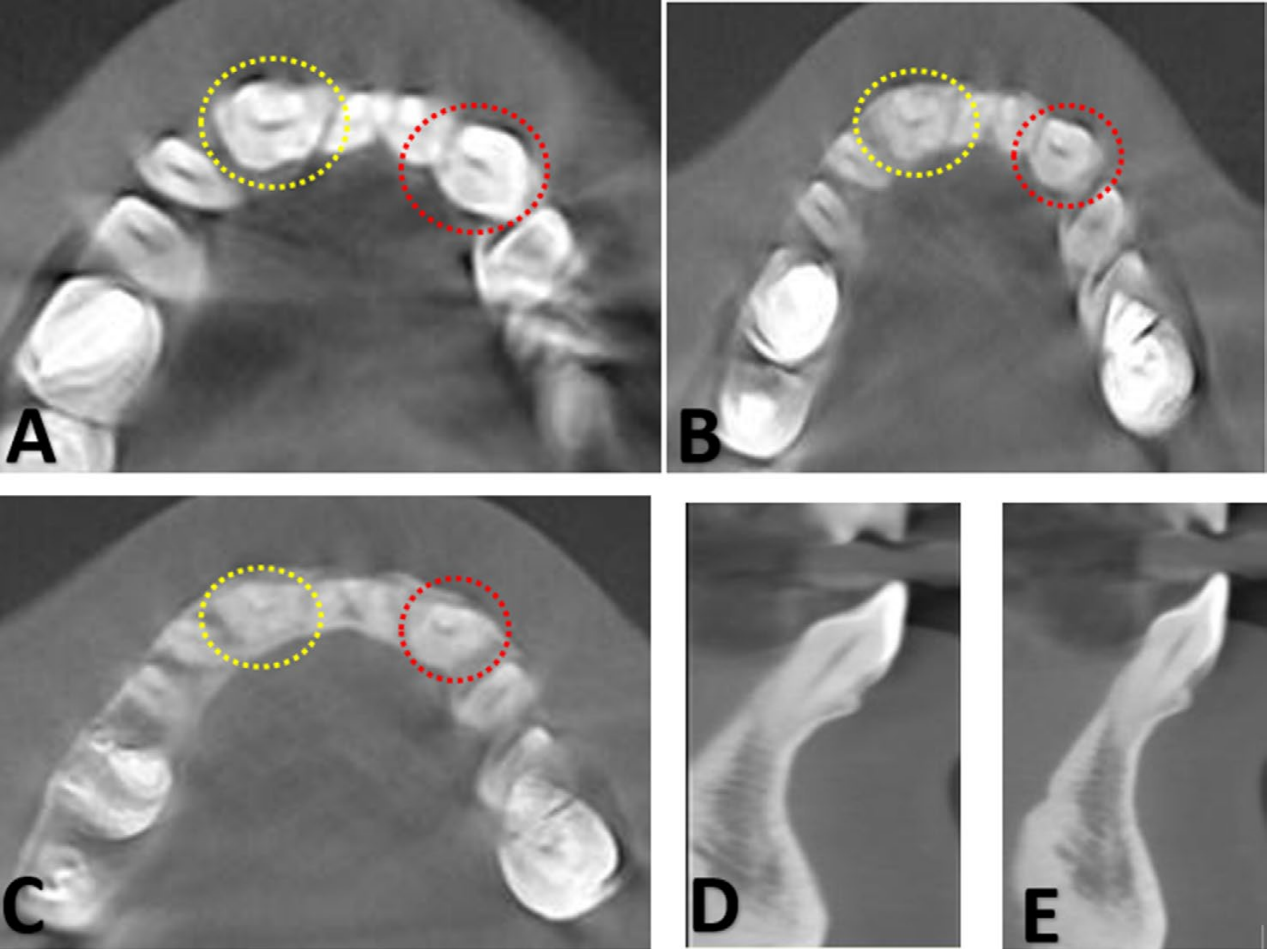
(A–C) show axial CBCT images at the cervical, middle, and apical thirds of the root, respectively, of the fused right and left lateral incisors and canines exhibiting a single pulp chamber and a single root. (D, E) show sagittal CBCT sections demonstrating similar crown and root morphology of the fused lateral incisor and canine on both sides. Minor image noise was noted, partially obscuring fine internal details of the root canals; nevertheless, the diagnostic interpretation remained unaffected.

Further assessment using cone‐beam computed tomography (CBCT) was performed to evaluate the morphology and pulpal anatomy. The axial CBCT images (Figure 2A–C) confirmed the presence of a single pulp chamber and a single root canal in both fused teeth. The sagittal sections (Figure 2D,E) revealed identical crown and root morphology, indicative of a bilaterally symmetrical presentation. Based on both clinical and radiographic findings, a complete bilateral fusion was diagnosed between the mandibular right lateral incisor and canine, characterized by a single root and a single pulp canal.

## 5 Discussion

The most common cause of fusion is physical contact between developing tooth germs, possibly resulting from external forces, pressure, or trauma during the morphodifferentiation stage of tooth development [15, 18]. Another proposed mechanism involves necrosis of the epithelial tissue separating the tooth germs, leading to fusion of the enamel organ and dental papilla. Fusion has been associated with various syndromes in the primary dentition, including chondroectodermal dysplasia, focal dermal hypoplasia, achondrodysplasia, median cleft facial syndrome, oral‐facial‐digital syndrome, otodental dysplasia, and Russell‐Silver syndrome [19].

Genetic factors also play a partial role in this anomaly. The identification of candidate gene variants such as *ERCC6*, *OBSCN*, *SLC27A3*, and *KIF25* suggests a potential genetic contribution to tooth fusion anomalies. However, it remains unclear whether these variants are associated with complete or incomplete fusion. The higher prevalence of fused teeth in Chinese and Mongoloid populations may be attributed to the increased frequency of variants such as ERCC6 (c.2204G>T) and SLC27A3 (c.1036C>T and c.1385G>A) in these groups [1]. These genetic associations remain preliminary and warrant further validation. Future studies employing molecular and developmental biology approaches such as functional analyses of gene expression during odontogenesis, animal models with targeted gene manipulation, and large‐scale genome‐wide association studies could provide valuable insights into how these variants influence dental tissue differentiation and morphodifferentiation. Understanding the molecular pathways involved may ultimately clarify the genetic mechanisms underlying tooth fusion and other developmental dental anomalies.

In the present case, symmetrical fusion likely resulted from the simultaneous contact between developing tooth buds on both sides of the lower jaw. The mandibular lateral incisors and canines were fused at the crown level, with a prominent labial groove. Each fused tooth exhibited a single root, suggesting that the fusion occurred before crown calcification, resulting in the complete fusion of both crown and root structures. The close proximity of tooth buds, along with their heightened sensitivity to developmental disturbances during the formative stage, may explain the relatively common occurrence of fusion in primary anterior teeth.

Based on the morphology and extent of fusion, Anguilo et al. classified fused teeth into four types [20, 21].

- Type I: bifid crown with a single root
- Type II: large crown with a large root
- Type III: two fused crowns with a double conical root
- Type IV: two fused crowns with two fused roots

Based on the above classification, the present case can be categorized as Type II on both sides, as clinical and radiographic examinations revealed a single large crown and root. Literature indicates that Type III is the most commonly occurring fusion type and Type IV is most susceptible to dental caries.

A literature review on the fusion of permanent mandibular teeth revealed one case of bilateral fusion involving the mandibular lateral incisor and canine showing incomplete fusion with separate roots and root canals on the right side, and complete fusion on the left [15]. Only two cases of unilateral fusion between the permanent mandibular lateral incisor and canine have been reported: one by Ayyildiz et al. in a 16‐year‐old male from Turkey [3] and another by Zhang et al. in a 37‐year‐old female from China [5]. Both cases exhibited complete fusion on the right side and lacked any significant medical or family history. Notably, these cases did not present with obvious clinical malocclusion such as crowding. In contrast, the present case showed evident crowding and difficulty in maintaining effective plaque control.

In primary teeth, fusion has been associated with an increased risk of dental caries, particularly in Type III fusion and it may affect the succeeding permanent teeth, leading to other dental anomalies such as fusion, peg‐shaped incisors and missing teeth [22]. Therefore, early detection is crucial to prevent potential adverse effects on the permanent dentition. In permanent teeth, fusion can lead to crowding, abnormal occlusion, and aesthetic concerns, as well as increased risks of dental caries, endodontic and periapical diseases, and periodontal issues [23].

Clinically, fusion must be distinguished from gemination, which occurs when a single tooth germ partially divides during development, resulting in a normal number of teeth in the arch. Radiographically, gemination typically presents with a single root and pulp chamber, along with two partially or completely separated crowns [4, 12]. Additionally, in gemination, the two halves of the crown are usually mirror images, whereas in fusion, the halves appear distinctly different [12]. Both gemination and fusion are more common in the primary dentition, with incisors being the most frequently affected.

Thorough clinical monitoring of fusion in both primary and permanent dentition is essential to prevent complications. In primary teeth, treatment focuses on preventive strategies to avoid caries until the permanent teeth erupt [24, 25]. In permanent teeth, malocclusion due to crowding or aesthetic concerns can be corrected with orthodontic treatment. In severe cases of crowding or periodontal pathology, surgical intervention may be necessary [26, 27].

In the present case, the patient exhibited noticeable crowding and difficulty in maintaining effective plaque control. Although orthodontic correction was recommended to address these issues, the patient declined treatment due to financial limitations. Therefore, the patient was placed on a regular follow‐up schedule every six months to evaluate plaque control, gingival health, and occlusal stability. Preventive measures included reinforcement of oral hygiene instructions, professional scaling and polishing at each visit, and topical fluoride application to strengthen enamel and reduce the risk of caries along the developmental grooves.

This report presents an exceptionally rare case of bilateral fusion of the permanent mandibular lateral incisors and canines in a 35‐year‐old female, resulting in crowding and complicating plaque control. Although orthodontic treatment was recommended to address aesthetic and functional concerns, the patient was unable to proceed due to financial limitations. She was educated on potential complications and advised to maintain meticulous oral hygiene and attend regular follow‐ups to monitor the fused teeth. This case highlights the importance of a thorough diagnostic approach, including detailed medical and dental history, comprehensive clinical examination, and radiographic assessment.

## Author Contributions

**Suresh Kandagal Veerabhadrappa:** conceptualization, data curation, investigation, methodology, project administration, writing – original draft, writing – review and editing. **Jayanth Kumar Vadivel:** writing – review and editing. **Mohamed Zahoor Ul Huqh:** writing – review and editing. **Siddharthan Selvaraj:** validation, visualization, writing – original draft, writing – review and editing. **Anand Marya:** supervision, validation, writing – review and editing. **Seema Yadav:** resources, software, supervision, validation, visualization, writing – original draft, writing – review and editing.

## Funding

The authors have nothing to report.

## Consent

Written informed consent was obtained from the patient for documentation and publication of any potentially identifiable images or data included in this article.

## Conflicts of Interest

The authors declare no conflicts of interest.

## Data Availability

Further data related to this case are available upon reasonable request directed to the corresponding author.
